# Supplementary figures and images for: A simple assay to quantify mycobacterial lipid antigen-specific T cell receptors in human tissues and blood
Source: PLoS Negl Trop Dis. 2021 Dec 16;15(12):e0010018. doi: 10.1371/journal.pntd.0010018 (PMC8717985; doi:10.1371/journal.pntd.0010018)

Supplemental Figure 1

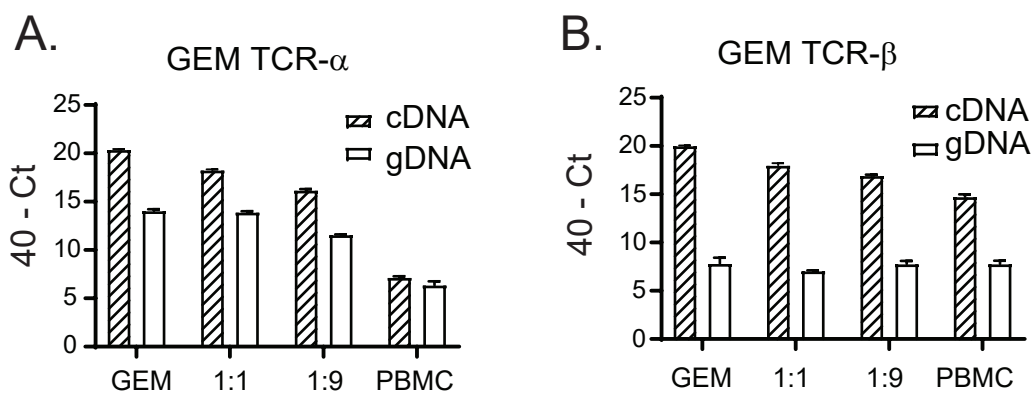

Supplement: S1 Fig — Expression of (A) GEM TCR-ɑ and (B) GEM TCR-β from GMM-specific T cell line (GEM) and PBMCs alone, and mixed in a 1:1 (GEM:PBMC) and 1:9 ratio using cDNA template vs gDNA template. (PDF) [file pntd.0010018.s001.pdf]
