# Supplementary material for: A simple assay to quantify mycobacterial lipid antigen-specific T cell receptors in human tissues and blood
Source: PLoS Negl Trop Dis. 2021 Dec 16;15(12):e0010018. doi: 10.1371/journal.pntd.0010018 (PMC8717985; doi:10.1371/journal.pntd.0010018)
Supplement: S1 Table — Human samples used in this study are listed by clinical cohort and sample identifier (Sample). The TB cohort (TB) is composed of individuals with pulmonary tuberculosis and either had positive sputum smear microscopy or positive culture for M. tuberculosis. Culture was only performed in individuals who had a negative sputum smear. The adolescent cohort study (ACS) is composed of individuals who were enrolled in a study to determine incidence of tuberculosis infection. Interferon-γ release assay (IGRA) and tuberculin skin test (TST) results are listed and concordantly positive or negative. (PDF) [file pntd.0010018.s002.pdf]

Supplemental Table S1

Clinical Cohort	Sample	age	sex	IGRA result	TST	sputum smear grade	Culture results
ACS	01-0345	14	M	negative	0	N/A	N/A
	01-0408	14	F	positive	10.4	N/A	N/A
	01-0470	15	F	positive	14.2	N/A	N/A
	01-0485	15	F	positive	14.5	N/A	N/A
	01-0606	16	F	negative	0	N/A	N/A
	01-0616	17	F	positive	17	N/A	N/A
	01-0667	15	F	positive	15.5	N/A	N/A
	01-0722	13	M	positive	17	N/A	N/A
	01-0924	18	M	positive	13	N/A	N/A
	02-0156	16	F	positive	14	N/A	N/A
	02-0184	17	M	negative	0	N/A	N/A
	02-0320	13	M	negative	0	N/A	N/A
	03-0203	18	F	positive	17	N/A	N/A
	03-0243	16	M	positive	17	N/A	N/A
	03-0295	12	F	positive	14.2	N/A	N/A
	03-0311	13	F	positive	18.5	N/A	N/A
	03-0324	12	M	negative	0	N/A	N/A
	03-0342	12	F	negative	0	N/A	N/A
	03-0539	12	M	positive	16.5	N/A	N/A
	03-0647	18	M	negative	0	N/A	N/A
	03-0703	17	F	negative	0	N/A	N/A
	03-0709	14	F	positive	16	N/A	N/A
	09-0092	13	M	positive	17	N/A	N/A
	09-0107	12	M	negative	0	N/A	N/A
	09-0157	13	M	negative	0	N/A	N/A
	09-0292	15	F	negative	0	N/A	N/A
	09-0428	13	F	positive	17	N/A	N/A
	09-0514	18	F	positive	12	N/A	N/A
	09-0809	17	M	positive	12	N/A	N/A
TB	TB-1100	36	F	N/A	N/A	3+	none*
	TB-1103	31	M	N/A	N/A	3+	none*
	TB-1104	30	M	N/A	N/A	1+	none*
	TB-1107	37	F	N/A	N/A	2+	none*
	TB-1108	21	F	N/A	N/A	2+	none*
	TB-1127	24	F	N/A	N/A	1+	none*
	TB-1129	29	F	N/A	N/A	3+	none*
	TB-1131	26	F	N/A	N/A	1+	none*
	TB-1136	35	F	N/A	N/A	Negative	Positive
	TB-1138	19	M	N/A	N/A	3+	none*

* Culture was not done; at the time in South Africa, a smear+ result was sufficient to diagnose TB/initiate anti-TB treatment, and sputum cultures were not routinely done in the public health clinics
