# Supplementary material for: A simple assay to quantify mycobacterial lipid antigen-specific T cell receptors in human tissues and blood
Source: PLoS Negl Trop Dis. 2021 Dec 16;15(12):e0010018. doi: 10.1371/journal.pntd.0010018 (PMC8717985; doi:10.1371/journal.pntd.0010018)
Supplement: S2 Table — The leprosy cohort is composed of individuals in Nepal who were enrolled based on clinical presentation of leprosy. Bacterial index represents the bacillary load in the biopsy sample. Comprehensive Ridley-Jopling (RJ) indicates how the individual was classified based on comprehensive assessment of all clinical and laboratory data. (PDF) [file pntd.0010018.s003.pdf]

Supplemental Table S2

Sample	age	sex	Biopsy Bacterial Index	Comprehensive RJ*
D-002	60	M	5	LL
D-005	35	F	Neg	BT
D-010	28	M	3	LL
D-013	60	M	6	LL
D-014	41	M	Neg	not leprosy
D-015	18	M	6	LL
D-018	29	M	Neg	TT
D-019	23	M	1	BL
D-020	47	M	6	LL
D-021	30	M	Neg	BT
D-023	39	F	1	BT
D-024	23	M	4	LL
D-025	18	M	Neg	TT
D-026	21	F	Neg	BT
D-028	19	M	4	LL
D-029	20	M	6	LL
D-032	21	F	Neg	not leprosy
D-037	19	M	Neg	not leprosy
D-042	25	F	4	BT
D-046	60	M	Neg	BT
D-047	37	M	5	LL
D-052	25	M	Neg	BT
D-057	38	F	Neg	BT
D-058	25	M	Neg	not leprosy
D-072	27	M	Neg	BT
D-074	33	M	5	BL
D-075	20	M	Neg	not leprosy
D-081	60	M	1	BT
D-083	53	F	Neg	BT
D-087	25	M	Neg	not leprosy
D-092	28	M	6	LL
D-095	26	M	Neg	TT
D-096	55	M	Neg	BT

*classified based upon comprehensive assessment of all clinical and lab data together
