# Supplementary material for: A simple assay to quantify mycobacterial lipid antigen-specific T cell receptors in human tissues and blood
Source: PLoS Negl Trop Dis. 2021 Dec 16;15(12):e0010018. doi: 10.1371/journal.pntd.0010018 (PMC8717985; doi:10.1371/journal.pntd.0010018)
Supplement: Attachment — Submitted filename: Response to Reviewers_final.docx [file pntd.0010018.s005.docx]

**Responses to Reviewer Comments**

**Methods**

- Are the objectives of the study clearly articulated with a clear testable hypothesis stated?
- Is the study design appropriate to address the stated objectives?
- Is the population clearly described and appropriate for the hypothesis being tested?
- Is the sample size sufficient to ensure adequate power to address the hypothesis being tested?
- Were correct statistical analysis used to support conclusions?
- Are there concerns about ethical or regulatory requirements being met?

**Reviewer #1: the description of the patient cohort is sufficient to address the questions raised in the manuscript. The sample sizes are not very high and the selection of samples is based on "availability" rather than prospective or clinically well-defined criteria. However this is considered and discussed adaquately. Ethical approvals were obtained.**

**Reviewer #2: (No Response)**

**Reviewer #3: Acceptable**

**Reviewer #4: (No Response)**

We thank the reviewers for their positive assessment of our work.

--------------------

**Results**

- Does the analysis presented match the analysis plan?
- Are the results clearly and completely presented?
- Are the figures (Tables, Images) of sufficient quality for clarity?

**Reviewer #1: see below**

**Reviewer #2: These features are okays presented**

**Reviewer #3: Acceptable**

**Reviewer #4: (No Response)**

Please see response to Reviewer #1 below.

--------------------

**Conclusions**

- Are the conclusions supported by the data presented?
- Are the limitations of analysis clearly described?
- Do the authors discuss how these data can be helpful to advance our understanding of the topic under study?
- Is public health relevance addressed?

**Reviewer #1: see below**

See response to Reviewer #1 below

**Reviewer #2: There is no significance of the data presented.**

We apologize for the confusion on this point. We have shown that mycobacterial lipid-specific T cells can be detected using a simple field-PCR assay. While the abundance of GEM-T cells did not differentiate between healthy and disease states in leprosy and tuberculosis, further advancements targeting more disease-specific T cells may allow for the development of simpler, field-friendly diagnostics for these diseases. We have modified the Discussion to emphasize this point:

Line 334 - 337: “Next generation assays might focus on lipids with more restricted expression. A natural candidate would be sulfoglycolipids, which are only expressed by virulent mycobacteria [24]. *An assay based on sulfoglycolipid-specific TCRs may better differentiate between healthy individuals and those with mycobacterial disease.*”

Line 383 - 387: “*While the abundance of GEM-T cells did not differentiate between healthy and disease states, further advancements targeting more disease-specific T cells may allow for the development of simpler, field-friendly diagnostics that would enable greater case detection of patients with TB and leprosy in clinical settings.*”

**Reviewer #3: Acceptable**

**Reviewer #4: (No Response)**

--------------------

**Editorial and Data Presentation Modifications**

- Use this section for editorial suggestions as well as relatively minor modifications of existing data that would enhance clarity. If the only modifications needed are minor and/or editorial, you may wish to recommend “Minor Revision” or “Accept”.

**Reviewer #1: (No Response)**

**Reviewer #2: The authors have developed a qualitative PCR assay for germline-encoded, mycolyl lipid-reactive (GEM) T cells, recognizes mycobacterial cell wall lipids, and expresses a conserved TCR-ɑ chain that is shared among genetically unrelated individuals. This assay was validated on plasmids and T cell lines. and tested on blood samples from South African subjects with or without tuberculin reactivity or with active tuberculosis disease. the authors were able to detect GEM TCR-ɑ above the limit of detection in 92% of donors but found no difference in GEM TCR-ɑ expression among the three groups after normalizing for total TCR-ɑ expression (Leprosy, TB diseased vs. healthy/exposed controls). This disappointing results decrease the utility of such a method.**

We agree that the GEM TCR-ɑ assay was not able to differentiate between healthy individuals and those with tuberculosis or leprosy. However, our data provided proof-of-concept that a simple assay is able to detect mycobacterial-specific TCR sequences from donor-unrestricted T cells above the limit of detection in both tissue and blood. Thus, our study may inspire further work to refine this technique using more disease specific TCR sequences. We have emphasized this in the Discussion:

Line 381 - 387: “As an alternative, we show that a simple real-time PCR based method focused on a single lipid-specific and donor-unrestricted TCR is feasible and yielded results above the limit of detection in blood and tissue specimens from all donors tested. *While the abundance of GEM-T cells did not differentiate between healthy and disease states, further advancements targeting more disease-specific T cells may allow for the development of simpler, field-friendly diagnostics that would enable greater case detection of patients with TB and leprosy in clinical settings.*”

**Reviewer #3: Accept**

**Reviewer #4: (No Response)**

--------------------

**Summary and General Comments**

- Use this section to provide overall comments, discuss strengths/weaknesses of the study, novelty, significance, general execution and scholarship. You may also include additional comments for the author, including concerns about dual publication, research ethics, or publication ethics. If requesting major revision, please articulate the new experiments that are needed.

**Reviewer #1: The major novelty of this study is the technical establishment of a molecular assay to measure lipid-specific T cell receptors in clinically relevant samples, including tissue biopsies. The description, presentation and interpretation of the results is very informative and clear. There are obvious limitations of the study: Limited sample size, analysis of two related, but distinct infectious diseases (rather than studying PBMC and biopsies (e.g. lymph nodes) from either tuberculosis or leprosy), no histology from the skin lesions to localize the cells within granulomas and confirm the PCR data). And of course the assay is not relevant for laboratory diagnosis of mycobacterial disease because it does not discrimate between healthy and infected individuals. All these points are critically addressed in the balanced discussion. Tthe authors are careful not to overinterpret the results and rather highlight the technical achievement (see title).**

We appreciate this positive assessment of our work.

**Reviewer #2: (No Response)**

**Reviewer #3: Zhou et al present an interesting study aimed to develop TCR-based molecular diagnostics of TB and Leprosy. Expanding technologies to timely diagnose neglected diseases like TB and Leprosy is an urgent public health need. The concept of applying TCR-based molecular diagnostics for the detection of infectious diseases has gained attraction with the detection of viral infection using classical MHC-restricted T cell responses against viral antigens. While T cell responses against Mtb immunodominant antigens using interferon-gamma release assays have been successfully applied to measure history of exposure to the bacteria, their utility in diagnosing complex diseases states in Leprosy and TB has been less clear, especially for rare CD1-reactive T cells, like GEM cells. These T cells have the advantage of bypassing HLA polymorphism, making this a logical and valuable direction to pursue. The authors thoroughly validate the diagnostic using plasmids and clones expressing the canonical TCR-alpha found in mycolyl-reactive CD1b-reactive T cell TCR rearrangements and apply it to samples from two cohorts to test this hypothesis. The authors acknowledge the limitation of the assay in distinguishing healthy vs. sick patients in either disease, suggesting limited antigen-specific clonal expansion in disease states. However, the novelty of this approach and rigor in analyzing the relative TCR abundance using valuable clinical cohorts is commendable, and open the concept of using DURTs to develop simple field diagnostics for further study.**

We appreciate this accurate summary and positive assessment of our work.

**Minor comments:**

**It is not clear from the figures if the authors had verified the lower number of N-nucleotides in GEM TRAV1-2-TRAJ-9 rearrangements by sequencing. Was that confirmed?**

The positive control plasmids containing the GEM TCR-ɑ and GEM TCR-β chains match those originally described by Van Rhijn et al. 2013, and the GMM-specific T cell line was sequenced as previously described (G10/CS110 in DeWitt et al. J immunol. 2018). We did not sequence the qPCR products to verify the lower number of N-nucleotides in GEM TRAV1-2-TRAJ-9 rearrangements in clinical samples. When we compared melt curves, we found that most of the samples contain a peak at 79°C similar to that seen with the positive control, suggesting that the sequences are identical (Fig. 4B). Notably, we were also able to detect other peaks, particularly around 76°C, suggesting amplification of non-canonical GEM TCR-ɑ sequences as well. We have modified the Methods and Results to reflect these details:

Line 143 - 147: “Generation of the GMM-specific T cell line *(G10)* used for assay validation were previously described [15]. *Briefly, PBMCs were isolated from healthy South African adults and incubated with GMM-loaded CD1b tetramer. Tetramer positive T cells were sorted using a FACSAria II (BD Biosciences) and expanded in culture. Cell lines were then screened using tetramer staining. Sequencing of TCRs was performed using the Immuno-SEQ assay (Adaptive Biotechnologies).*”

Line 292 - 295: “We found that there was a peak around the expected temperature of 79~~.3~~°C *in most samples, suggesting sequences identical to our plasmid and cell line positive controls*, but ~~some samples~~ others also contained ~~a~~ secondary peak~~s~~, *particularly around 76°C* (Figure 4B). *This suggests possible amplification of non-canonical GEM TCR-ɑ sequences.*”

**In figure 2C, the relative expression of GEM TCR-beta appears to be a hundred-fold lower than GEM TCR-alpha. How do you explain that? I would expect that the GEM TCR-alpha rearrangement to be far less abundant than the GEM TCR-beta counterparts.**

We apologize for the confusion. The y-axis in figure 2C is measuring relative expression of the GEM TCR-ɑ/β in GMM-specific T cells compared to their expression in PBMCs. The difference in magnitude in the two graphs is likely due to non-specificity of the GEM TCR-β primers, and therefore greater abundance of sequences detected in PBMCs, rather than any true difference between expression of GEM TCR-ɑ and GEM TCR-β in any of the samples. We have edited the results and figure legend of Figure 2C to clarify this.

Line 241 - 244: “We observed a similar pattern for GEM TCR-β, though the relative decrease in expression in PBMC samples was less than that observed for the GEM TCR-ɑ sequence (Figure 2A). This is especially evident *when comparing total TCR-normalized expression of GEM TCR in the GMM-specific T cell line relative to PBMCs* ~~after normalizing expression to total TCR levels~~ (Figure 2C).”

Line 258 - 260: “(C). Relative expression of GEM TCR-ɑ and GEM TCR-β in a GMM-specific T cell line, and mixed GEM:PBMC populations, compared to expression in PBMCs alone. Expression of GEM TCR-ɑ and GEM TCR-β was normalized to total TCR abundance. ~~Relative expression of GEM TCR-a and GEM TCR-b after normalization to total TCR abundance.~~”

**The analysis of lepromatous and tuberculoid leprosy legions is intriguing and important considering that it is ultimately the site of disease in leprosy. Since very little is known about the homing properties of CD1b-reactive T cells, do you hypothesize that CD1b/GMM-reactive T cells would preferentially home to the lesions in a similar manner to CD1a-reactive T cells in autoinflammatory conditions like dermatitis? Please comment in the discussion.**

We appreciate this comment and question. Briefly, our data on dermal biopsies from individuals with leprosy show that the abundance of GEM T cells are increased in the skin compared to controls, though it is unclear if they are preferentially recruited as the proportion of total T cells are also increased. There is evidence from previous studies that CD1b is upregulated in inflammatory lesions in the skin in individuals with tuberculoid leprosy and after *B. burgdorferi* infection, suggesting the possibility for specific recruitment to the skin during infection [Sieling et al. J immunol. 1999, Yakimchuk et al. Eu J Immunol. 2011]. We have added the following text and references to the Discussion to emphasize this point.

Line 349-360: “*The homing properties of CD1b-restricted T cells are not well-defined. However, CD1a-restricted T cells are known to home to the skin and have been implicated in various skin conditions [Genardi et al. J Inv Derm. 2021]. For instance, CD1a-restricted T cells have been shown to express cutaneous lymphocyte antigen, and could be isolated from human skin biopsies and produce IL-22 after activation [de Jong et al. Nat Immunol. 2010, Cotton et al. J clin invest. 2021]. CD1a-restricted T cells from blood and/or skin also recognize antigens implicated in the pathogenesis of skin diseases including psoriasis [Cheung et al. J Exp Med. 2016] and atopic dermatitis [Jarrett et al. Sci Tranl Med. 2016], as well as in wasp and bee venom responses [Bourgeois et al. J Exp Med. 2015, Subramaniam et al Eur J Immunol. 2016]. While CD1a is constitutively expressed on epidermal Langerhan cells, CD1b is normally rare in the skin, but has shown to be upregulated in the dermis after infection with Borrelia burgdorferi [Yakimchuk et al. Eu J Immunol. 2011] Previous studies have also shown increased expression of CD1b proteins in dermal granulomas of individuals with tuberculoid leprosy [Sieling et al. J immunol. 1999] and human pulmonary TB granulomas [Chancellor et al. PNAS. 2017] raising the potential for recruitment of CD1b-restricted T cells to sites of infection.*”

References that were added:

1. Genardi et al. J Inv Derm. 2021 PMID: 34130802
2. de Jong et al. Nat Immunol. 2010 PMID: 21037579,
3. Cotton et al. J clin invest. 2021 PMID: 33393500
4. Cheung et al. J Exp Med. 2016 PMID: 27670592
5. Jarrett et al. Sci Tranl Med. 2016 PMID: 26865566
6. Bourgeois et al. J Exp Med. 2015 PMID: 25584012
7. Subramaniam et al Eur J Immunol. 2016 PMID: 26518614
8. Yakimchuk et al. Eu J Immunol. 2011 PMID: 21246541
9. Sieling et al. J immunol. 1999 PMID: 9973451
10. Chancellor et al. PNAS. 2017 PMID: 29158404

**Reviewer #4:**

**1. Findings looks intersecting and novel approach for the current sittings, but still few things need to be consider from the current approach**

**2. Did authors able to define a sensitivity and specificity for the developed assays.**

We appreciate this question. Using histopathology as the gold standard for leprosy diagnosis and IGRA for TB, our assay was unable to differentiate between individuals with or without mycobacterial disease. Thus, we were not able to calculate sensitivity or specificity of the assay. We have added the following text to the Discussion to emphasize this limitation:

Line 325 - 327: “We found that the abundance of GEM TCR-ɑ did not distinguish among mycobacterial disease, infection, or control populations*, limiting its use as a diagnostic assay*.”

**3. Why do authors think even in the IGRA negative samples GEM-TCRa able to detect more than the limit of detection.**

This is an excellent question. The GEM TCR mediated recognition of mycolic acid and GMM, which are produced non-tuberculous mycobacteria and other members of the Actinomyetales family. Therefore, it is possible that while individuals were not previously exposed to *M. tuberculosis*, they show evidence of T cell priming to GMM or mycolic acid antigen as a result of exposure to these other bacteria.

Our result is consistent with studies using lipid-loaded tetramers to identify GMM- or MA-specific T cells via flow cytometry. Analysis of blood samples collected from individuals in South Africa showed that frequencies of GEM T cells were not different between adolescents who tested IGRA-positive and IGRA-negative. [Layton. J Immunol. 2018]. A study performed in Peru showed that frequencies of GMM-specific or MA-specific T cells were not more abundant in individuals who were IGRA positive or had active tuberculosis disease when compared to IGRA-negative controls. [Lopez et al. Front Immunol. 2020]. We have modified the Discussion to emphasize this context for our work:

Line 328 - 334: “*In addition,* though GMM is preferentially produced in the context of infection, both mycolic acid and GMM are present in non-tuberculous mycobacteria and other members of the family Actinomyetales as well as *M. tuberculosis* and *M. leprae* [19,22,23]. Thus, it is possible that GMM-specific T cell responses in control subjects were primed by exposure to environmental mycobacteria. *This may be one of the reasons we were able to detect GEM TCR-ɑ sequences in individuals who tested IGRA negative.*“

Line 362 - 365: “Although earlier research suggested that GEM-T cells expand in mycobacterial infection [10,15], our data is consistent with recent findings that frequencies of CD1b-GMM or CD1b-MA-specific T cells detected using flow cytometry are not higher in individuals with active TB or a positive IGRA compared to *individuals who were IGRA negative* ~~control subjects~~ [11,39].”

**4. Authors can also discuss few points about the rationale for this study.**

Our T cell receptor repertoires are generated by our history of antigenic exposure. This property allows for diagnosis of infectious diseases through measurement of disease-specific TCRs using deep-sequencing and advanced computational analysis [Emerson et al. Nat Genet. 2017, Elyanow et al. medRxiv. 2021]. Because mycobacterial antigen-specific T cells are conserved even across unrelated individuals, we hypothesized that we could measure their frequencies with a qPCR assay, which could allow for development of simpler, field-friendly assays for mycobacterial disease. We have detailed this rationale for our work in the Introduction.

Line 87 - 97: “In addition to foreign peptide antigens, T cells have also evolved to recognize bacterial lipid antigens via the highly conserved CD1 antigen presenting system [9]. A unique feature of CD1-restricted T cells is that they sometimes express T cell receptors whose sequence is conserved across genetically unrelated individuals. For example, some T cells that recognize mycolic acid derivatives, which are abundant in the mycobacterial cell wall, always express TCR-ɑ chains containing a rearrangement of the TCR-ɑ variable (TRAV)-1-2 and TCR-ɑ joining (TRAJ) 9 genes [10]. These cells are known as germline encoded mycolyl-reactive (GEM) T cells and are detectable at high-frequency in the blood of humans with latent and active TB as well as non-human primates after vaccination with BCG [11,12]. Because the GEM TCR-ɑ sequence is conserved across genetically unrelated individuals, quantitative detection should be possible without the need for deep-sequencing or advanced computational methods.”

**5. Please add a detailed demographic profile of the study population as a table in the manuscript.**

We have added several Supplementary Tables that now detail the demographic and clinical characteristics of the cohorts. Supp. Table 1 list the age, sex, sputum smear grade, and sputum culture results for individuals with active TB, as well as the age, sex, IGRA and TST results for the individuals tested for latent TB. Supp Table 2 list age, sex, bacterial biopsy index and Ridley-Joplin scores for samples from individuals from Nepal with and without leprosy.

**6. Did all the active TB patients are pulmonary TB cases?**

All active TB patients had either positive sputum smear or sputum culture for M.tb (see new Supp Table 1).

**7. Since the samples size per group is very low it has to be mentioned in the study limitations.**

We have added the following text to the Discussion to emphasize this point:

Line 327 - 328: *“Our overall sample sizes were low, potentially limiting our ability to detect small differences.*”

**8. Please do add AFB smear grade status and culture results status in the demographic tables.**

We have made this change in the new Supp. Table 1.

**9. Authors should discuss few points how this results can be used in the clinical settings or patient management.**

Though our specific assay cannot diagnose individuals with mycobacterial disease, the results provide proof-of-concept that such a field-deployable diagnostic assay could be realized with further improvements in our method using disease-specific TCRs. We have updated the Discussion to emphasize this point.

Line 383 - 387: “*While the abundance of GEM-T cells did not differentiate between healthy and disease states, further advancements targeting more disease-specific T cells may allow for the development of simpler, field-friendly diagnostics that would enable greater case detection of patients with TB and leprosy in clinical settings.”*

--------------------

**PLOS authors have the option to publish the peer review history of their article. If published, this will include your full peer review and any attached files. If you choose “no”, your identity will remain anonymous but your review may still be made public. Do you want your identity to be public for this peer review?**

**Reviewer #1: No**

**Reviewer #2: No**

**Reviewer #3: Yes: Sara Suliman**

Thank you, Dr. Suliman, for your thoughtful comments! They have certainly improved the manuscript.

**Reviewer #4: No**

--------------------

**Additional comments:**

When gathering updated demographic information, we noted that a sample previously listed as “not leprosy” in our Nepal clinical cohort was now classified as “borderline tuberculoid”. Given this, we reclassified that sample for our study and created updated Figures 4A & C. We have also modified the methods, results, and discussion sections to reflect this change.

Line 187 - 190: “Based on availability of cDNA, we selected ~~7~~ *6* samples from leprosy negative individuals, 12 samples from individuals with lepromatous leprosy (10 lepromatous, 2 borderline lepromatous), and ~~14~~ *15* from individuals with tuberculoid leprosy (3 tuberculoid, ~~11~~ *12* borderline tuberculoid) for our experiments.”

Line 289 - 290: “In all samples tested, we were able to detect expression of the GEM TCR-ɑ above the limit of detection, and this was ~~consistently~~ higher in *lepromatous* leprosy patients compared to controls subjects (Figure 4A, right).”

Line 342 - 346: “We found an increased abundance of the GEM TCR-ɑ sequence in dermal biopsies from individuals with lepromatous ~~and tuberculoid~~ leprosy compared to control subjects. This increase was correlated with an increase in total TCR-ɑ abundance *in both lepromatous and tuberculoid leprosy. There was a trend towards increase in GEM TCR-ɑ in the skin of individuals with tuberculoid leprosy, though this did not reach statistical significance, possibly due to small sample sizes.*”

**Editorial Comments**

**Figure Files**:

**While revising your submission, please upload your figure files to the Preflight Analysis and Conversion Engine (PACE) digital diagnostic tool,** [**https://pacev2.apexcovantage.com**](https://pacev2.apexcovantage.com/)**. PACE helps ensure that figures meet PLOS requirements. To use PACE, you must first register as a user. Then, login and navigate to the UPLOAD tab, where you will find detailed instructions on how to use the tool. If you encounter any issues or have any questions when using PACE, please email us at** [**figures@plos.org**](mailto:figures@plos.org)**.**

COMPLETED

**Data Requirements**:

**Please note that, as a condition of publication, PLOS' data policy requires that you make available all data used to draw the conclusions outlined in your manuscript. Data must be deposited in an appropriate repository, included within the body of the manuscript, or uploaded as supporting information. This includes all numerical values that were used to generate graphs, histograms etc.. For an example see here:** [**http://www.plosbiology.org/article/info%3Adoi%2F10.1371%2Fjournal.pbio.1001908#s5**](http://www.plosbiology.org/article/info%3Adoi%2F10.1371%2Fjournal.pbio.1001908#s5)**.**

We have created an excel document with spreadsheets containing the data used to generate the corresponding manuscript figures. See Data Supplement 1.

**Reproducibility**:

**To enhance the reproducibility of your results, we recommend that you deposit your laboratory protocols in protocols.io, where a protocol can be assigned its own identifier (DOI) such that it can be cited independently in the future. Additionally, PLOS ONE offers an option to publish peer-reviewed clinical study protocols. Read more information on sharing protocols at** [**https://plos.org/protocols?utm_medium=editorial-email&utm_source=authorletters&utm_campaign=protocols**](https://plos.org/protocols?utm_medium=editorial-email&utm_source=authorletters&utm_campaign=protocols)

We have updated our methods to include all thermocycler conditions used. Our methods also detail the kits and primers used for our assays.

Line 116-118: “Thermocycler conditions included denaturation at 50°C *for 2 min* and 95°C *for 2 min*, followed by 40 cycles at 95°C for 15 s and 60°C for 1 min. Melt curves were generated by increasing temperatures 0.3°C/s from 60°C to 95°C.”

**References**

**Please review your reference list to ensure that it is complete and correct. If you have cited papers that have been retracted, please include the rationale for doing so in the manuscript text, or remove these references and replace them with relevant current references. Any changes to the reference list should be mentioned in the rebuttal letter that accompanies your revised manuscript. If you need to cite a retracted article, indicate the article's retracted status in the References list and also include a citation and full reference for the retraction notice.**

COMPLETED, see added references listed above.
